# CCR6^+^ Th cell distribution differentiates systemic lupus erythematosus patients based on anti-dsDNA antibody status

**DOI:** 10.7717/peerj.4294

**Published:** 2018-02-09

**Authors:** Wei Zhong, Zhenyu Jiang, Jiang Wu, Yanfang Jiang, Ling Zhao

**Affiliations:** 1 Department of Rheumatology, First Hospital, Jilin University, Changchun, China; 2 College of Electrical Engineering and Instrumentation, Jilin University, Changchun, China; 3 Genetic Diagnosis Center, First Hospital, Jilin University, Changchun, China; 4 Key Laboratory of Zoonosis Research, Ministry of Education, First Hospital, Jilin University, Changchun, China

**Keywords:** Systemic lupus erythematosus, CCR6^+^ T helper cells, Anti-dsDNA antibody

## Abstract

**Background:**

Systemic lupus erythematosus (SLE) disease has been shown to be associated with the generation of multiple auto-antibodies. Among these, anti-dsDNA antibodies (anti-DNAs) are specific and play a pathogenic role in SLE. Indeed, anti-DNA^+^ SLE patients display a worse disease course. The generation of these pathogenic anti-DNAs has been attributed to the interaction between aberrant T helper (Th) cells and autoimmune B cells. Thus, in this study we have investigated whether CCR6^+^Th cells have the ability to differentiate SLE patients based on anti-DNA status, and if their distribution has any correlation with disease activity.

**Methods:**

We recruited 25 anti-DNA^+^ and 25 anti-DNA^−^ treatment-naive onset SLE patients, matched for various clinical characteristics in our nested matched case-control study. CCR6^+^ Th cells and their additional subsets were analyzed in each patient by flow cytometry.

**Results:**

Anti-DNA^+^ SLE patients specifically had a higher percentage of Th cells expressing CCR6 and CXCR3. Further analysis of CCR6^+^ Th cell subsets showed that anti-DNA^+^ SLE patients had elevated proportions of Th9, Th17, Th17.1 and CCR4/CXCR3 double-negative (DN) cells. However, the proportions of CCR6^−^ Th subsets, including Th1 and Th2 cells, did not show any association with anti-DNA status. Finally, we identified a correlation between CCR6^+^ Th subsets and clinical indicators, specifically in anti-DNA^+^ SLE patients.

**Conclusions:**

Our data indicated that CCR6^+^ Th cells and their subsets were elevated and correlated with disease activity in anti-DNA^+^ SLE patients. We speculated that CCR6^+^ Th cells may contribute to distinct disease severity in anti-DNA^+^ SLE patients.

## Introduction

Systemic lupus erythematosus (SLE) is a multi-organ autoimMune disorder that can affect the skin, joints, kidneys and central nervous system (Kaul et al., 2016). These effects are usually mediated by generation of auto-antibodies and immune complexes. Indeed, SLE has been observed to be associated with more than 100 different auto-antibodies including anti-nuclear antibody (ANA), anti-dsDNA antibody (anti-DNA), anti-Sm antibody, anti RNP antibody, anti Ro antibody, anti La, and anti-phospholipid antibody (Sherer et al., 2004). Among these, anti-DNA is a crucial pathogenic SLE factor and its production leads to SLE development (Kaul et al., 2016). In addition, it is one of the 11 criterion used for SLE diagnosis and has been observed to play a prominent role in SLE, as highlighted by the observation that two-thirds of SLE patients have detectable anti-DNA (Fattal et al., 2014) levels. The identification of anti-DNA in SLE patients very early—and even before the onset of disease—further highlights its role in a clinically overt disease (Arbuckle et al., 2003; Kaul et al., 2016). Typically, anti-DNA^+^ SLE patients display a severe disease course, and suffer from significant type III/IV lupus nephritis (LN) (Conti et al., 2015; Mavragani et al., 2015). These patients also have worse treatment outcomes in comparison to anti-DNA^−^ patients (Lazarus et al., 2012; Linnik et al., 2005). One study has demonstrated a direct link between the development of anti-DNA and the HLA-DRB1 ∗ 1501(DR2) allele polymorphism in SLE patients (Podrebarac, Boisert & Goldstein, 1998), implying a breakdown of immunological tolerance against self-antigens, most likely through aberrant MHC class II dependent activation of auto-reactive CD4^+^ T cells. During normal immune response to foreign antigens, there is inherently a production of pathogenic auto-antibodies in the germinal centers of follicles (Ray, Putterman & Diamond, 1996). A particular population of autoimmune CD4^+^ T cells usually drive the production of pathogenic anti-DNA by cognate interactions with the autoimmune B cells found in SLE (Datta, 1998). In SLE patients, aberrant T cell activation, coupled with nonspecific activation of B cells, leads to overproduction of a plethora of auto-antibodies. Specifically, pathogenic anti-DNA are of an IgG type and are used as a quintessential clinical biomarker. These findings also suggest that anti-DNA-producing B cells have undergone isotype switching, dependent upon the presence of T cells (Pisetsky, 2016).

Generally T cells and in particular CD4^+^ cells initiate and perpetuate autoimmunity. Infiltrating activated T cells have been observed in tissue samples collected from organs affected by lupus (Crispin et al., 2008; Enghard et al., 2009; Robak et al., 2001). In addition, cytokines expressed by T cells, including TNF-*α* and IL-17, both of which play an active role in autoimmunity, are increased in SLE patients (Vincent et al., 2013). Moreover, the chemokine receptor, CCR6, expressed on Th cells, has been implicated in mediating the recruitment of IL-17 producing cells in glomerulonephritis (Koga et al., 2016; Turner et al., 2010).

Such chemokine receptors have typically been used to characterize memory Th cell subsets, with different effector and migratory functions (Sallusto, Mackay & Lanzavecchia, 2000). Due to heterogeneity, CCR6^+^ Th cell can be distinguished into several subpopulations, such as IL-17A and IL-22 producing CCR6^+^ T cell subpopulations. CCR6^+^ cells with Th17 characteristics have a CCR4^+^CCR10^−^CXCR3^−^ phenotype (Duhen et al., 2009; Trifari et al., 2009; Van Hamburg et al., 2013), while those with Th22 characteristics display a CCR4^+^CCR10^+^ phenotype (Duhen et al., 2009; Mahnke, Beddall & Roederer, 2013). Interestingly, Th17.1 cells with CCR6^+^CCR4^−^CXCR3^+^ phenotype can produce both IL-17 and IFN-*γ*, which were previously thought to be mutually exclusive functional characteristics (Acosta-Rodriguez et al., 2007). In addition, IL-9 producing Th9 cells, which have CCR6^+^CCR4^−^ phenotype, has been suggested to play a role in wound healing of pleural mesothelial cells during *M. tuberculosis* infection (Ye et al., 2012). However, CCR6^−^ Th cells, Th1 cells with CCR6^−^CCR4 ^−^CCR10^−^CXCR3^+^ phenotype and producing IFN-*γ* (Bonecchi et al., 1998; Duhen et al., 2009), and Th2 cells with CCR6^−^CCR4^+^CXCR3^−^ phenotype, are involved in secreting IL-5, IL-4 and IL-13 chemokines (Rivino et al., 2004). Based on the differential expression of CCR6 on Th cells, recent studies have indicated their potential proinflammatory role in the development of autoimmune disorders, including rheumatoid arthritis (Paulissen et al., 2015a; Paulissen et al., 2015b). It has also been demonstrated that pathogenic Th17 cells expressing CCR6 play a key role in accelerating organ injury in animal models of glomerulonephritis (Turner et al., 2010) and arthritis (Hirota et al., 2007). In addition, a genetic link has also been reported between CCR6 gene polymorphisms and LN susceptibility (Zhou et al., 2015).

Thus, crucial role of anti-DNA in SLE pathogenesis, association of its production with T cell engagement, its involvement along with CCR6^+^ Th cells in kidney impairment, and differential clinical course between anti-DNA positive and negative patients, prompted us to investigate the differences in Th cell distribution between anti-DNA^+^ and anti-DNA^−^ SLE patients. In addition, we also tested the possibility of a correlation between Th cell subsets and disease activity in anti-DNA^+^ SLE patients.

## Materials and Methods

### Patients and samples

We performed a matched case-control study where 25 anti-DNA^+^ and 25 anti-DNA^−^ treatment-naive onset SLE patients were recruited from the in-patient service of the First Hospital of Jilin University, China between January 2016 and January 2017. All patients had clinical symptoms for less than three months. Patients were matched for SLE disease activity score, presence of anti-Sm antibodies, sex, age, and disease duration. SLE diagnosis was conducted according to the revised criteria for the classification of SLE by the American College of Rheumatology (Hochberg, 1997; Tan et al., 1982), and the disease activity of each patient was assessed based on the SLE disease activity index (SLEDAI) (Bombardier et al., 1992). Complicated LN in the patients with renal involvement was defined according to the ACR criteria, where patient typically displayed: (i) persistent urinary protein level of ≥0.5 g/day; (ii) presence of active cellular casts; and (iii) biopsy evidence of lupus nephritis (Hahn et al., 2012; Schiffenbauer & Simon, 2004). However, subjects with a history of systemic sclerosis, myositis or other autoimmune diseases, or had a recent infection or received immunosuppressive or glucocorticoid treatment within the past six months, were excluded. In addition, written informed consent was obtained from individual participants. Authors had access to information that could identify individual participants during and after data collection. The experimental protocol was established based on the Declaration of Helsinki guidelines, and was approved by the Human Ethics Committee of Jilin University, China (approval number: 2016-373).

### Data collection

The demographic and clinical characteristics of the subjects, including age and gender at baseline, were obtained from the hospital records. Routine laboratory tests, including determination of complete blood cell counts, concentrations of plasma complement factors (C3, C4), erythrocyte sedimentation rate (ESR), C-reactive protein (CRP) were measured as described previously (Zhao et al., 2013). The levels of serum anti-DNA and anti-Sm antibody were measured by indirect immunofluorescence using special kits, according to the instruction of manufacturers (Oumeng, Beijing, China).

### Isolation of peripheral blood mononuclear cells (PBMCs)

The peripheral blood (5 mL) was collected from all participants after an overnight fasting, and PBMCs were subsequently isolated by density-gradient centrifugation at 800 g for 30 min using Ficoll-Paque Plus (Amersham Biosciences, Buckinghamshire, UK). The cells were later resuspended at a concentration of 1 × 10^6^ per mL in RPMI-1640 culture medium (Invitrogen, Carlsbad, CA, USA) containing 10% fetal calf plasma.

### Flow cytometry analysis

The PBMCs were stained in duplicate with APC-H7-anti-CD4 (Becton Dickinson, San Diego, CA, USA), PerCP-Cy5.5-anti-CD3 (Becton Dickinson), BV510-anti-CCR4 (Becton Dickinson), PE-Cy7-anti-CCR6 (Becton Dickinson), BB515-anti-CCR10 (Becton Dickinson), and PE-CF594-anti-CXCR3 (Becton Dickinson) antibodies in the dark at 4 °C for 30 min. Negative controls were stained with isotype-matched control antibodies (APC-H7-anti-IgG1, PerCP-Cy5.5-anti-IgG1, BV510-anti-IgG1, PE-Cy7-anti-IgG1, BB515-anti-IgG2a, PE-CF594-anti-IgG1). The frequencies of different T cell subsets were examined by flow cytometry analysis on a FACSAria II (Beckton Dickinson, San Diego, CA, USA) and analyzed by FlowJo software for Microsoft (v7.6.2, TreeStar, San Carlos, CA, USA).

### Statistical analysis

Statistical analysis of the data was performed using SPSS 21.0 (SPSS, Chicago, IL, USA) software. The quantitative data were represented as individual values, or median (range) of each group. The differences between two groups of patients were analyzed using Wilcoxon matched-pairs signed-ranks test. The correlation analysis was performed using Spearman’s rank correlation test. A two-sided *P* value of <0.05 represented statistical significance.

## Results

### Demographic and clinical characterization of SLE patients based on anti-DNA status

A total of 25 SLE patients with anti-DNA positive status and another 25 matched patients with negative anti-DNA status were recruited to undertake this study. Their demographic and clinical characteristics are summarized in Table 1. Specifically, anti-DNA^+^ SLE patients displayed lower concentrations of C3 and C4 proteins (*P* = 0.0010, *P* = 0.0150). However, a higher number of anti-DNA^+^ SLE patients showed renal impairment (*P* = 0.0460), thereby indicating higher activation of the complement system and subsequent worse prognosis. Interestingly, many of the other parameters studied in these patients showed no significant differences based on anti-DNA status.

**Table 1:** Demographic and clinical characteristics of anti-DNA^+^ and anti-DNA^−^ treatment-naive patients with onset SLE.

Parameters	anti-DNA^+^	anti-DNA^−^
	(*n* = 25)	(*n* = 25)
Age (years)	31 (18–61)	27 (17–58)
Gender: female/male	21/4	21/4
SLEDAI	17 (2–36)	19 (7–39)
Positive anti-Sm	14 (56.00%)	10 (40.00%)
ESR (mm/h)	46 (8–90)	55 (3–118)
CRP (mg/L)	9.40 (0.43–51)	6.85 (3.16–192)
C3 (IU/mL)	0.36 (0.12–1.52)^*^	0.73 (0.17–1.62)
C4 (U/mL)	0.06 (0.00–0.35)^*^	0.15 (0.02–0.50)
Renal involvement	16 (64%)^*^	8 (32%)

**Notes.**

Data shown are median (range) or number of cases.

SLE systemic lupus erythematosus anti-DNA anti-dsDNA antibodies SLEDAI SLE disease activity index ESR Erythrocyte sedimentation rate CRP C-reactive protein. Normal values: ESR: 0–15 mm/h, CRP: 0–3 mg/L, C3: 0.9–1.8 units/mL,C4: 0.1–0.4 units/mL

* *P* < 0.05 versus anti-DNA^−^ SLE.

### Anti-DNA^+^ SLE patients revealed increased percentages of CCR6^+^ and CXCR3^+^ Th cells

**Figure 1:**
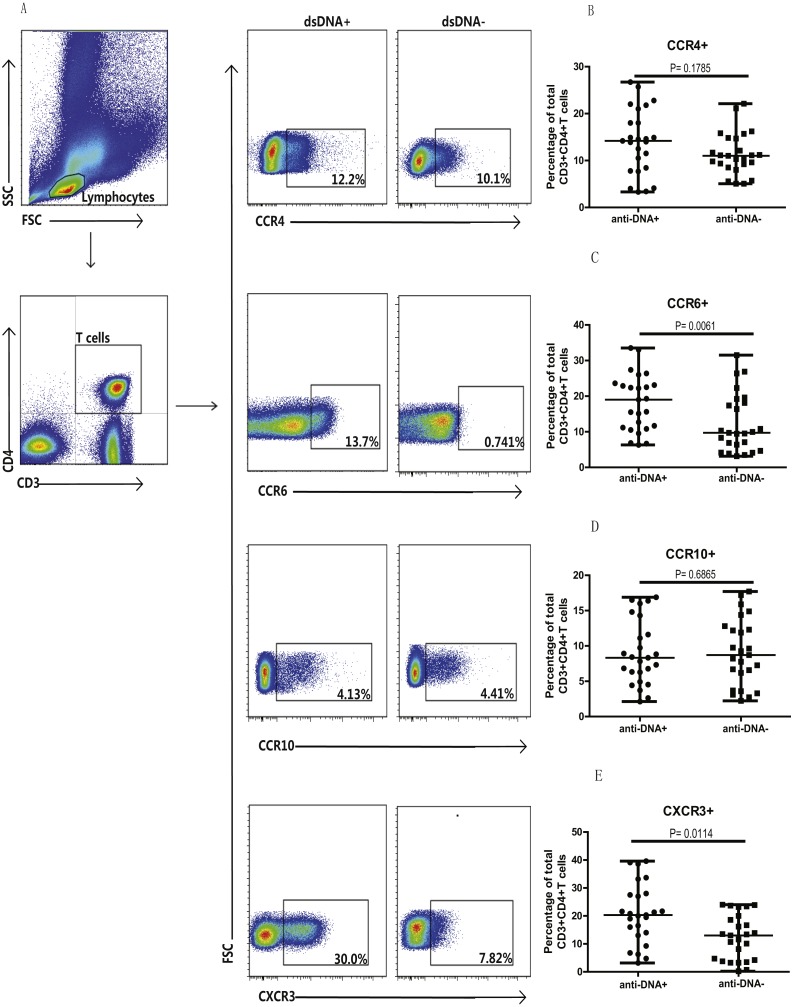
Analysis of circulating CD3^+^ & CD4^+^ T cells based on chemokine expression in SLE patients. Peripheral blood mononuclear cells (PBMCs) collected from new onset anti-DNA^+^ and anti-DNA^−^ SLE patients at baseline (*n* = 25 for each group) were analyzed by flow cytometry. (A) represented the gating strategy, while (B–E) showed the percentages of CCR4^+^, CCR6^+^, CCR10^+^ and CXCR3^+^ Th cells, respectively in anti-DNA^+^ and anti-DNA^−^ SLE patients.

Next, we analyzed the Th cell population in SLE patients from both groups, based on chemokine expression profile, as different chemokine receptors are expressed on the surface of T cells. Specifically, the expression of CCR4, CCR6, CCR10 and CXCR3 chemokines was analyzed on T cells (CD3^+^CD4^+^) isolated from peripheral blood mononuclear cells (PBMCs) of these patients. Flow cytometry analysis illustrated significantly higher proportions of CCR6^+^ (C) (*P* = 0.0061) and CXCR3^+^ (E) (*P* = 0.0114) T cells in anti-DNA^+^ SLE patients in comparison to anti-DNA^−^ SLE patients. However, no significant differences were observed in Th cells expressing CCR4 (B) (*P* = 0.1785) and CCR10 (D) (*P* = 0.6865) between these two groups of patients, as shown in Fig. 1. In addition, percentage of total CCR6^+^ Th cells correlated positively with anti-DNA titer in SLE patients (*r* = 0.4668  *P* = 0.0006; Table 2), but no correlations were observed between anti-DNA titers and CCR4^+^, CCR10^+^, CXCR3^+^ T cells (*P* > 0.05; Table 2).

**Table 2:** Correlation of Th cell populations (%CD3^+^CD4^+^ T cells) with anti-DNA titers in SLE patients.

Populations	*r*	*P* value
CCR4^+^ T cells	0.1734	0.2286
CCR6^+^ T cells^*^	0.4668	0.0006
CCR10^+^ T cells	0.0221	0.8789
CXCR3^+^ T cells	0.2729	0.0552
Th1 cells	0.2449	0.0865
Th2 cells	0.0478	0.7406
Th9 cells^*^	0.4218	0.0023
Th17 cells^*^	0.3608	0.0101
Th17.1 cells^*^	0.4192	0.0024
Th22 cells	0.0682	0.6377
CCR6^+^CCR4^+^CXCR3^+^ T cells	0.1972	0.1700
CCR6^+^CCR4^−^CXCR3^−^ T cells^*^	0.4510	0.0010

**Notes.**

SLE systemic lupus erythematosus anti-DNA anti-dsDNA antibodies

All analyses were performed using Spearman’s rank correlation test.

* *P* < 0.05.

### Anti-DNA^+^ SLE patients specifically showed elevated proportions of CCR6^+^ Th9, Th17, Th17.1 and CCR6^+^CCR4^−^CXCR3^−^ Th cells subsets

**Figure 2:**
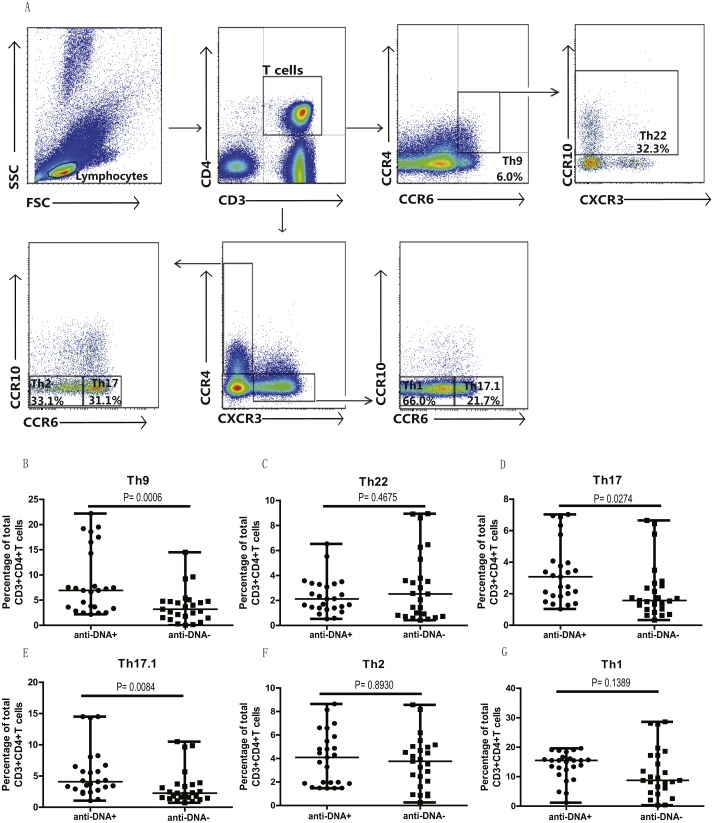
Analysis of the percentages of various circulating Th cell subsets in SLE patients. PBMCs collected from new onset anti-DNA^+^ and anti-DNA^−^ SLE patients (*n* = 25 in each group) at baseline were analyzed by flow cytometry for the percentages of different Th subset. (A) depicted the gating strategy for these different Th subsets, while (B)–(G) showed the comparative quantitative analysis of Th9 (CCR6^+^CCR4^−^), Th22 (CCR6^+^CCR4^+^CCR10^+^), Th17 (CCR6^+^CCR4^+^CXCR3^−^CCR10^−^), Th17.1 (CCR6^+^CCR4^−^CXCR3^+^CCR10^−^), Th2 (CCR6^−^CCR4^+^CXCR3^−^CCR10^−^), and Th1 (CCR6^−^CCR4^−^CXCR3^+^CCR10^−^) subsets in anti-DNA^+^ and anti-DNA^−^ SLE patients.

**Figure 3:**
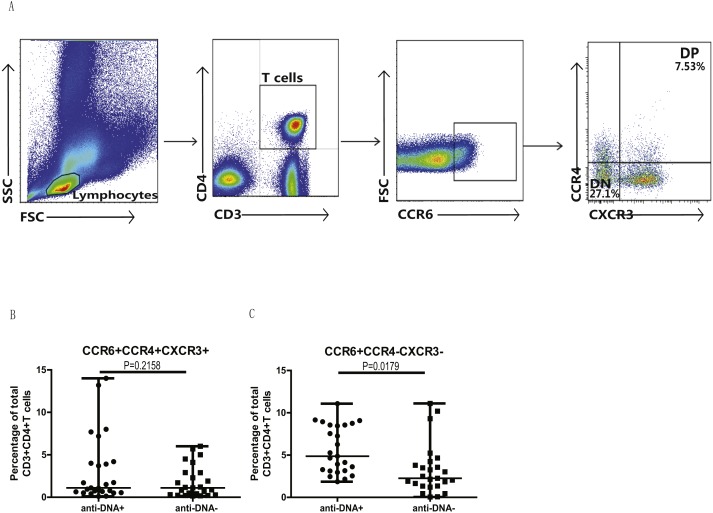
Analysis of circulating CCR4/CXCR3 DP and CCR4/CXCR3^−^ DN CCR6^+^ T cells in SLE patients. PBMCs collected from new onset anti-DNA^+^ and anti-DNA^−^ SLE patients (*n* = 25 for each group) at baseline were analyzed by flow cytometry for the percentages of unclassified CCR6^+^ Th subsets, CCR6^+^CCR4^+^CXCR3^+^, double positive(DP) and CCR6^+^CCR4^−^CXCR3^−^, double negative (DN) Th cells. (A) represented the gating strategy, while (B) and (C) showed the comparative quantitation of these Th cell subsets in anti-DNA^+^ and anti-DNA^−^ SLE patients.

In addition, we also analyzed the different subsets of Th cells based on the enrichment of different chemokine expression in SLE patients from both groups. CD3^+^CD4^+^ T cells were gated based on chemokine expression to identify Th1, Th2, Th9, Th17, Th22 or a Th17.1 phenotypes, as shown in Fig. 2A. CCR6^+^CCR4 ^+^CCR10^+^ phenotype depicted Th22 cells, while CCR6 ^+^CCR4^+^CXCR3^−^CCR10^−^ phenotype represented Th17 cells, and CCR6^+^ CCR4^−^ phenotype established Th9 cell subset. Also, Th17.1 cells with Th1 and Th17 profile were gated as CCR6^+^CCR4^−^CXCR3^+^CCR10^−^. In addition, we also measured the percentages of unclassified CCR6^+^ T cells, including CCR4/CXCR3 double-positive (DP) (CCR6^+^CCR4^+^CXCR3^+^ phenotype) and double-negative (DN) (CCR6^+^CCR4^−^CXCR3^−^ phenotype) Th cells, as shown in Fig. 3A. Our overall analysis of these different Th cell subsets in SLE patients based on anti-DNA status indicated that CCR6^+^ Th subsets, such as Th9 (*P* = 0.0006) (Fig. 2B), Th17 (*P* = 0.0274) (Fig. 2D), Th17.1 (*P* = 0.0084) (Fig. 2E) and CCR6^+^CCR4^−^CXCR3^−^ Th cells (*P* = 0.0179) (Fig. 3C), were significantly increased in anti-DNA^+^SLE patients. However, no significant difference was observed in the distribution of CCR6^+^ Th22 (*P* = 0.4675) (Fig. 2C) and CCR6^+^CCR4^+^CXCR3^+^ Th cells (*P* = 0.2158) (Fig. 3B) between anti-DNA^+^ and anti-DNA^−^ SLE patients. Positive correlations of anti-DNA titer were observed with Th9 (*r* = 0.4218, *P* = 0.0023), Th17 (*r* = 0.3608 *P* = 0.0101), Th17.1 (*r* = 0.4192 *P* = 0.0024) and CCR6^+^CCR4^−^CXCR3^−^ Th (*r* = 0.4510 *P* = 0.0010; Table 2) cells in SLE patients. Similarly, CCR6^−^ Th subpopulations, such as Th1 (*P* = 0.1389) (Fig. 2G) and Th2 (*P* = 0.8930) (Fig. 2F), which were gated as CCR6^−^CCR4^−^CXCR3^+^CCR10^−^ and CCR6^−^CCR4^+^CXCR3^−^CCR10^−^, respectively (Fig. 2A), did not show any significant differences between patients of both groups, and also showed no correlation with anti-DNA titer in these patients (*P* > 0.05; Table 2).

### Elevated percentage of CCR6^+^ Th9 subsets correlated with anti-DNA status in SLE patients

**Figure 4:**
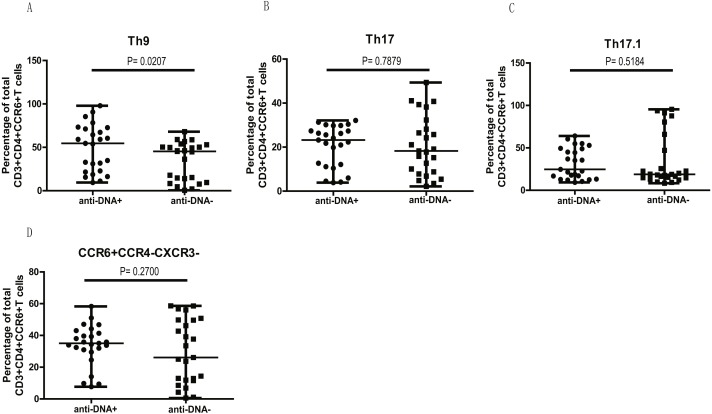
Comparative analysis of the circulating Th9, Th17, Th17.1 and CCR6^+^CCR4^−^CXCR3^−^ Th subsets in SLE patients. The quantitative analysis of the percentages of circulating Th9, Th17, Th17.1 and CCR6^+^CCR4^−^CXCR3^−^ Th cells in anti-DNA^+^ and anti-DNA^−^ SLE patients.

We next examined if higher levels of CCR6^+^ Th9, Th17, Th17.1 and CCR4/CXCR3 DN Th cell populations in anti-DNA^+^ SLE patients were due to the overall increase of CCR6^+^ Th cells (Fig. 1C) or whether they specifically correlate with anti-DNA status. Thus, we plotted all CCR6^+^ Th cell subpopulations as a proportion of total CCR6^+^ Th cells in each group. Interestingly, the proportions of all CCR6^+^ subpopulations were comparable between anti-DNA^+^ and anti-DNA^−^ patients, except Th9 cells (A) (*P* = 0.0207), which were significantly higher in anti-DNA^+^ patients than anti-DNA^−^ patients, as shown in Fig. 4. This observation indicated that elevated Th9 cell subset might have some functional correlation with anti-DNA status.

### Clinical indicators correlated with CCR6^+^ Th cell population in anti-DNA^+^ SLE patients

**Figure 5:**
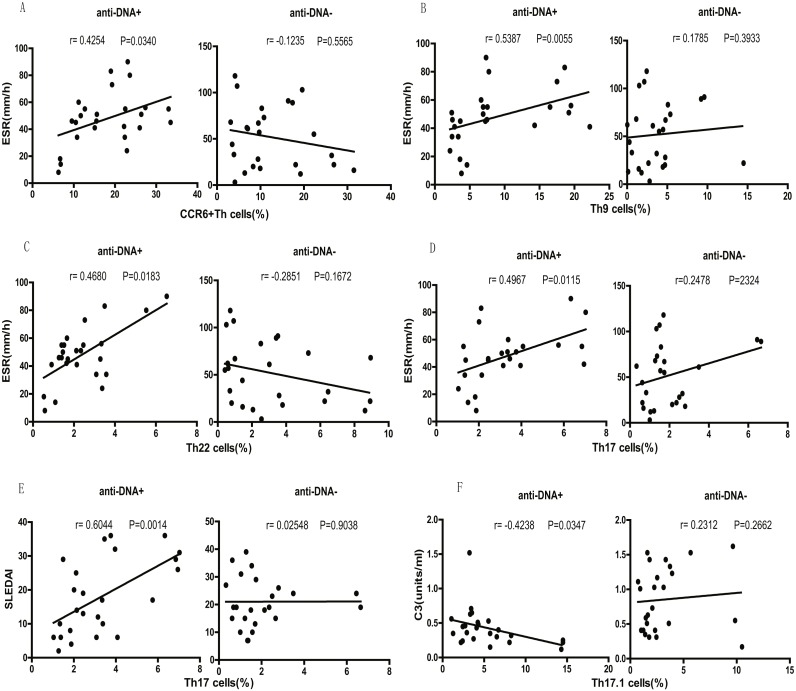
Correlational analysis between circulating CCR6^+^ Th subsets and clinical parameters of anti-DNA^+^ and anti-DNA^−^ patients. (A–D) depicted the correlation analysis of ESR value with circulating CCR6^+^ Th cells, Th9 cells, Th22 cells and Th17 cells respectively in anti-DNA^+^ and anti-DNA^−^ SLE patients. (E) showed the similar correlation between SLEDAI value and the percentage of circulating Th17 cells, while (F) represented the correlation between C3 value and the percentage of circulating Th17.1 cells. All analyses were performed using Spearman’s rank correlation test.

Finally, we tested the correlation between different SLE clinical indicators (disease activity) and CCR6^+^ Th cell subsets. We observed that the percentage of total CCR6^+^ Th cells correlated positively with ESR (*r* = 0.4254  *P* = 0.0340; Fig. 5A) in anti-DNA^+^ SLE patients. Further analysis of the correlation of specific subsets of CCR6^+^ Th cell with clinical parameters suggested that Th9 (*r* = 0.5387  *P* = 0.0055; Fig. 5B), Th22 (*r* = 0.4680  *P* = 0.0183; Fig. 5C), Th17 (*r* = 0.4967 *P* = 0.0115; Fig. 5D), CCR6^+^CCR4/CXCR3 DP (*r* = 0.4438 *P* = 0.0262; Fig. S1A), and CCR6^+^CCR4/CXCR3 DN (*r* = 0.6383 *P* = 0.0006; Fig. S1C) subsets positively correlated with ESR. In addition, Th17 cells (*r* = 0.6044  *P* = 0.0014; Fig. 5E), CCR6^+^CCR4/CXCR3 DP (*r* = 0.7740 *P* < 0.0001; Fig. S1B), and CCR6^+^CCR4/CXCR3 DN (*r* = 0.6453 *P* = 0.0005; Fig. S1D) subsets also showed positive correlation with SLEDAI. Interestingly, Th17.1 cell subset numbers showed an inverse correlation with C3 levels (*r* =  − 0.4238  *P* = 0.0347; Fig. 5F). Importantly, the anti-DNA^−^ SLE patients revealed no significant correlation between the frequency of CCR6^+^ Th cell subsets and any disease indicator (*P* > 0.05; Fig. 5 and Fig. S1).

## Discussion

In this study, we have specifically focused to assess the distribution of CCR6^+^ Th cells in anti-DNA^+^ SLE patients. Our results clearly demonstrated that anti-DNA^+^ onset SLE patients have higher levels of peripheral CCR6^+^ Th cells in comparison to anti-DNA^−^ patients. Further analysis of additional Th cell subsets confirmed elevated percentages of Th9, Th7, Th17.1 and CCR4/CXCR3 DN CCR6^+^ Th cells. Interestingly, these cell subsets showed positive correlations of anti-DNA titers in SLE patients. However, we identified that the elevated percentages in most of the Th cell subsets were due to an elevated proportion of total CCR6^+^ Th cells, except for Th9 subset. This specific observation highlighted the importance of Th9 cell subset in anti-DNA^+^ SLE patients. In addition, the percentages of CCR4/CXCR3 DP CCR6^+^ Th and Th22 cells were similar in anti-DNA^+^ and anti-DNA^−^ SLE patients. However, our findings revealed an increase in the percentage of Th1 and Th2 cells in anti-DNA^+^ SLE patients in comparison to anti-DNA^−^ patients. However, this difference was not statistically significant. Importantly, anti-DNA^+^ SLE patients specifically showed a correlation between the percentages of CCR6 ^+^ Th cells and their subsets and clinical indicators. However, anti-DNA^−^ patients showed no correlation between clinical indicators and CCR6^+^ or CCR6^−^ Th cells.

Thus, our data suggested that the distribution of CCR6^+^ Th cell populations correlates with anti-DNA status, and is consistent with previously published studies, which linked anti-DNA^+^ status of SLE patients with Th cells (Datta, 1998; Pisetsky, 2016; Podrebarac, Boisert & Goldstein, 1998; Ray, Putterman & Diamond, 1996). Anti-DNA positivity has also been shown to be strongly associated with MHC class II-restricted HLA-DRB1 ∗1501(DR2) allele, and anti-DNAs are synthesized due to cognate interactions between aberrantly activated T cell and autoimmune B cells. Thus, one can speculate that specific T cells might play a functional role in anti-DNA^+^SLE patients (Mohan et al., 1993). In this context, we found a positive correlation between the elevated percentage of CCR6^+^ Th populations and ESR in anti-DNA^+^ SLE patients, thereby supporting the notion that these cells indeed may play a critical role in disease activity specifically, in anti-DNA^+^ SLE patients. It is important to highlight that the correlation of CCR6^+^ Th cells with disease activity was not observed in anti-DNA^−^ SLE patients.

A significant association between CCR6 gene polymorphism and susceptibility to LN in SLE patients has also been reported (Zhou et al., 2015). Furthermore, it has been identified that CCR6 chemokine expressed by the effector/memory CD4^+^ T cells plays a role tissue damage in SLE patients, including LN or neuropsychiatric SLE patients, and probably functions through CCR6/CCL20 axis at the inflamed sites (Koga et al., 2016). These observations tentatively indicate that CCR6^+^ Th cells might be engaged in the aggravation of inflammatory reactions, and thus contributes to worse disease outcomes in anti-DNA^+^ SLE patients. Higher levels of CCR6^+^ T cells might play a role in risk-stratification of SLE patients, especially those with positive anti-DNA. It is well established that CCR6^+^ Th subpopulations display considerable heterogeneity and various subpopulations can be identified based on the differential expression of chemokine receptors. However, the contribution of these CCR6^+^ Th subsets in the disease severity of anti-DNA^+^ SLE patients has not been fully investigated.

Among various CCR6^+^ Th cell subsets, we identified that Th9 cells might be functionally important in anti-DNA^+^ SLE patients. These cells are derived from naive T cells in the presence of cytokines, thymic stromal lymphopoietin (TSLP), transforming growth factor (TGF)-β and IL-4, and would also require interferon regulatory factor-4 (IRF-4) and PU.1 transcription factors (Staudt et al., 2010; Veldhoen et al., 2008). In SLE patients, there have been few studies that have explored the role of Th9 cells along with its related cytokine IL-9, and have indicated an increase in Th9 cells and IL-9 serum levels in SLE patients in comparison to healthy controls (Dantas et al., 2015; Ouyang et al., 2013), along with a positive correlation with SLEDAI (Ouyang et al., 2013). Therefore, this indicated that Th9 subpopulation might play a role in SLE. Furthermore, using MRL/lpr mice (an animal model of lupus), it has been demonstrated that Th9 cells and IL-9 levels expand in the spleen and kidney of these mice, and neutralizing IL-9 antibody lead to diminished anti-DNA titers and alleviation of LN (Yang et al., 2015). Consistent with these observations, our study also showed that elevated frequencies of Th9 cells showed a positive correlation with ESR in anti-DNA^+^ SLE patients, while in anti-DNA^−^ SLE patients, Th9 cells showed no correlation with any of the clinical indicators.

Another CCR6^+^ Th cell subset which has been shown to be significantly elevated in SLE patients was Th17 cell subset, along with cytokine IL-17, and had been suggested to play a critical role in the development of SLE (Garrett-Sinha, John & Gaffen, 2008; Zhang et al., 2016). Consistent with this study, we also observed an increase in the levels of Th17 cells in anti-DNA^+^ SLE patients. This correlation could be accounted for due to the activation of NLRP3 by anti-DNA (Shin et al., 2013; Zhang et al., 2016), which in turn promotes Th17 cell differentiation (Bruchard et al., 2013), and thus contributed to disease severity by expanding pathogenic Th17 cells (Zhang et al., 2016). However, due to the high plasticity of Th17 cells, they often shift rapidly into a Th1-like phenotype, resulting in the production of IL-17 and IFN-y tand hence are also referred as Th17/Th1, non-classic Th1 (Cosmi et al., 2014), or Th17.1 cells (Paulissen et al., 2015b). It has also been shown that Th17.1 cells can be polarized from from naïve T cells directly, in *C. albicans*-primed cultures (Zielinski et al., 2012). Th17.1 cells appear to be more pathogenic than Th17 in human inflammatory disorders (Cosmi et al., 2014), and express transporter protein multi-drug resistance type 1 (MDR1), which results in insensitivity to glucocorticoids (Ramesh et al., 2014). Elevation of Th17.1 cells levels have been observed in anti-citrullinated protein antibodies (ACPA) positive RA patients with worse disease outcome, than in ACPA negative RA patients (Paulissen et al., 2015b). Notably, we also observed higher levels of Th17.1 cells and their negative correlation with C3 protein levels in anti-DNA ^+^ SLE patients, thereby suggesting that Th17.1 probably contribute to a higher disease activity and worse treatment outcomes in anti-DNA^+^ SLE patients.

Interestingly, we also observed increased frequencies of CCR4/CXCR3 DN and CCR6^+^ T cells in anti-DNA^+^ SLE patients and a correlation of different clinical parameters with the CCR4/CXCR3 DN and DP CCR6^+^ T cells. DP cells usually show both Th1 and Th17 characteristics, while DN cells have Th17 cell specific features with expression of RORC and CD161 proteins (Paulissen et al., 2015a). In RA patients, these DP and DN CCR6^+^ Th subsets have been shown to manifest a higher activating effect on synovial fibroblasts than Th1 and naive cells (Paulissen et al., 2015a). However, their roles in SLE remain unclear and require further analysis.

In addition, CCR6^+^ T cells also included several additional cell subsets, like CCR6^+^ Tregs that might play a role in tissue-protection, and has been reported to be elevated during active SLE (Schmidt et al., 2017). Therefore, appearance of CCR6^+^ T cells might just reflect compensatory/protective immunologic reaction provoked by more severe disease in anti-DNA^+^ patients. Thus, additional studies are warranted to clarify the actual function of these Tregs, as well as the relevance of CCR6 coexpression in different T cell subsets.

## Conclusions

Overall, our data suggested that CCR6^+^ Th cells may contribute to disease severity in anti-DNA^+^ SLE patients, as they were specifically elevated in these patients and showed a correlation with ESR. In addition, our results also led us to propose that CCR6^+^ Th cells may serve as a prognostic indicator for risk-stratification and may prove a novel therapeutic target for the treatment of SLE. It would, therefore, be interesting to further characterize the dynamic role played by these cells specifically in anti-DNA^+^ SLE patients, using a larger sample size.

##  Supplemental Information

10.7717/peerj.4294/supp-1 Figure S1 Correlational analysis between circulating unclassified CCR6^+^ Th subsets and clinical parameters of anti-DNA^+^ and anti-DNA^−^ patients All analyses were performed using Spearman’s rank correlation test.

10.7717/peerj.4294/supp-2 Data S1 Raw data of the study Gender: 1 refers to male, 2 refers to female; antidsDNA: 0 refers to negative, 1 refers to positive; antiSM: 0 refers to negative, 1 refers to positive; dsDNA: levels of antidsDNA antibodies.

## Additional Information and Declarations

### Data Availability

The following information was supplied regarding data availability: The raw data has been provided as a Supplemental File.
