# Supplementary figures and images for: CCR6+ Th cell distribution differentiates systemic lupus erythematosus patients based on anti-dsDNA antibody status
Source: PeerJ. 2018 Feb 9;6:e4294. doi: 10.7717/peerj.4294 (PMC5808313; doi:10.7717/peerj.4294)

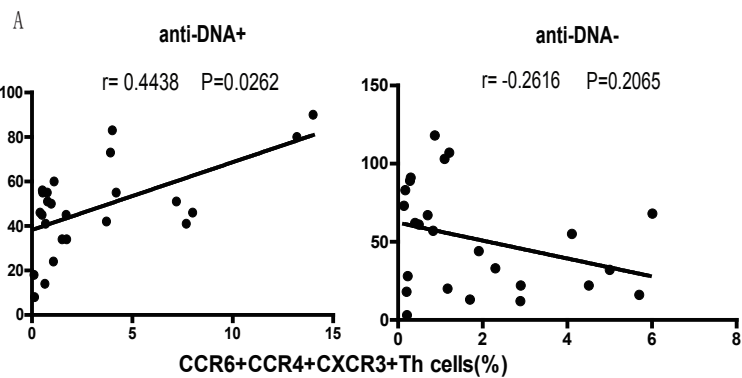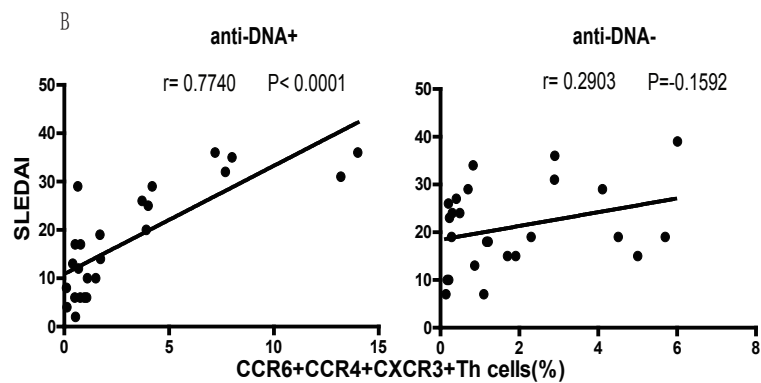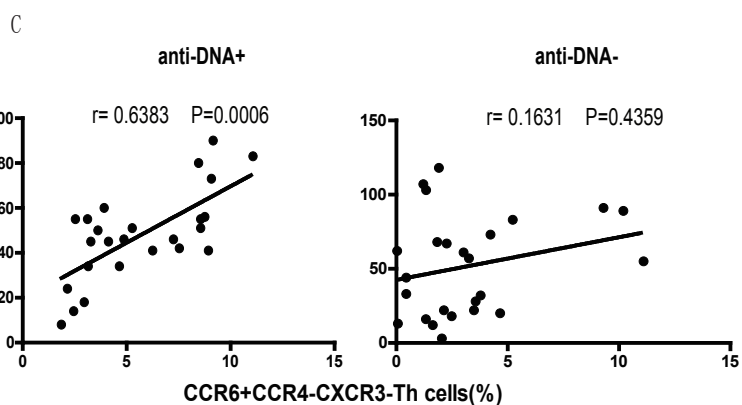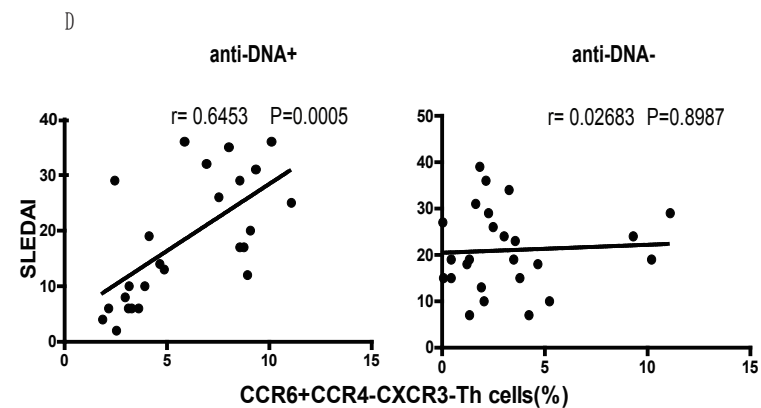

Supplement: Figure S1 — All analyses were performed using Spearman’s rank correlation test. [file peerj-06-4294-s001.pdf]
